# Comprehensive Extraction and Chemical Characterization of Bioactive Compounds in Tepals of *Crocus sativus* L.

**DOI:** 10.3390/molecules28165976

**Published:** 2023-08-09

**Authors:** Fabrizio Ruggieri, Maria Anna Maggi, Michela Rossi, Roberto Consonni

**Affiliations:** 1 Dipartimento di Scienze Fisiche e Chimiche, Università degli Studi dell’Aquila, Via Vetoio, Coppito, 67100 L’Aquila, Italy; fabrizio.ruggieri@univaq.it (F.R.); m.maggi@hortusnovus.it (M.A.M.);; 2 National Research Council, Institute of Chemical Sciences and Technologies “G. Natta” (SCITEC), Via Corti 12, 20133 Milan, Italy

**Keywords:** saffron tepals, kaempferol and anthocyanins, antioxidant, extraction of bioactive compounds

## Abstract

*Crocus sativus* L. is largely cultivated because it is the source of saffron, a well-appreciated and valued spice, not only for its culinary use but also because of its significant biological activities. Stigmas are the main product obtained from flowers, but in addition, tepals, largely considered a waste product, represent a big source of flavonoids and anthocyanins. This study aimed to delve into the phytochemical composition of saffron tepals and investigate whether the composition was influenced by the extraction technique while investigating the main analytical techniques most suitable for the characterization of tepal extracts. The research focuses on flavonoids, a class of secondary metabolites, and their health benefits, including antioxidant, anti-inflammatory, and anticancer properties. Flavonoids occur as aglycones and glycosides and are classified into various classes, such as flavones, flavonols, and flavanones. The most abundant flavonoids in tepals are kaempferol glycosides, followed by quercetin and isorhamnetin glycosides. Overall, this review provides valuable insights into the potential uses of tepals as a source of bioactive compounds and their applications in various fields, promoting a circular and sustainable economy in saffron cultivation and processing.

## 1. Introduction

*C. sativus* L. is an autumnal herbaceous flowering plant belonging to the Iridaceae family. It is a perennial meadow grass grown from its bulbs and reaches 10 to 25 cm in height. It is largely cultivated (80% of the world production) in Iran (Khorasan and Farse province), India (5% of the world production in Pulwama and Budgam districts), Afghanistan (Pashtun Zarghun district), Greece (Western Macedonia), Morocco (Marrakesh and east of Taroudant), Spain (Castile-La Mancha), and Italy (Sardinia, Abruzzo). Other countries have lower production and have recently started to increase their cultivation (Turkey, France, Switzerland, Pakistan, China, Japan, and Australia) due to the low agronomical requirement and high economic value.

**Figure 1:**
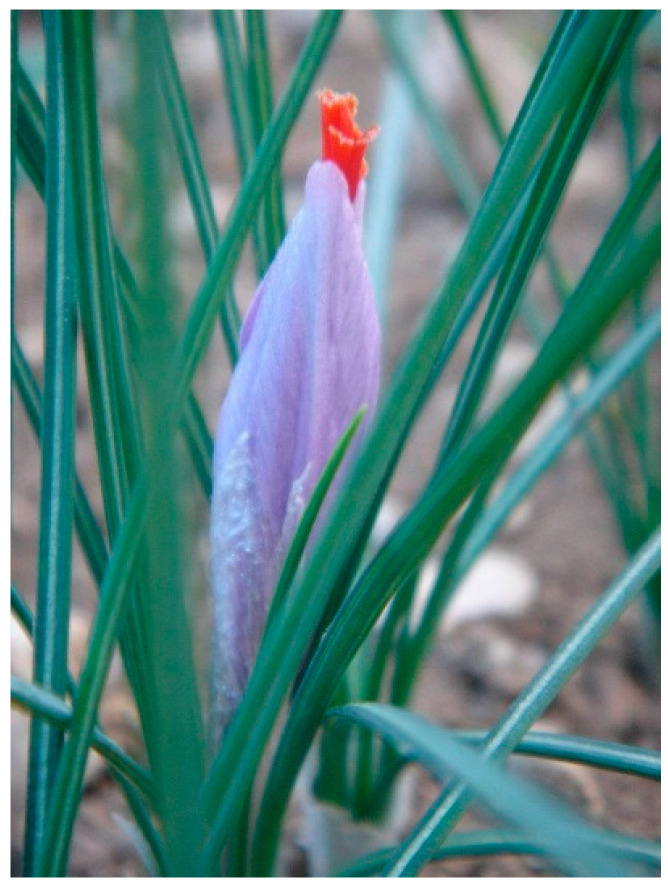
Saffron flower before harvest.

The flowers, typically one up to three per bulb, have six petaloid tepals, three on the inner side (sepals or stamens) and three on the outer side (petals) joined with a long pipe that comes out from the bulb (Figure 1). The most important parts of the flower are the stigmas, three of them for each flower, which are manually collected and further subjected to a different drying processes according to regional/national practices—this process gives rise to the spice called saffron. This valuable spice is distributed by producers worldwide and is typically used as a culinary condiment. In addition, saffron is endowed with several pharmacological activities reported in both preclinical and clinical studies. The clinical efficacy of saffron has been checked against different pathologies, such as diabetes [1], age-related macular degeneration [2,3], cognitive impairment [4,5,6,7], glaucoma [8], sexual dysfunction in women [9] and men [10], and premenstrual syndrome [11]. However, most studies assessed its antidepressant activity, and its efficacy against mild or moderate depression has been confirmed by a recent meta-analysis [12]. In fact, stigmas contain several characteristic secondary metabolites, including carotenoids such as crocetin and crocins (the glycosylated form of crocetin), picrocrocin (a glycosylated monoterpenoid), and safranal (the corresponding aldehydic moiety of picrocrocin aglycone).

The other parts of the flower, stamens (filament and anthers) and tepals, are generally considered by-products and remain unused even though they consist of a huge amount of plant material with respect to stigmas. As a matter of fact, to obtain only 1 kg of dried stigmas, approximately 63 kg of flowers, 53 kg of tepals, 1500 kg of leaves, 100 kg of spathes, and hundreds of bulbs too small and/or having physical or biological alterations are generally discarded. Tepals are typically used as fertilizer for the soil or as ornamentation of dishes/products based on saffron. Tepals are a source of protein, fiber, fats, and essential minerals (K, Ca, and P) necessary for the growth of animals. Therefore, they can be used as animal feeding sources [13]. Interestingly, Ghanbari [14] showed that mycorrhizal fungus inoculation, combined with fertilizer, improved saffron productivity, stigma quality, and total phenolic and flavonoid contents in tepals [15].

The content of available biomolecules in the leaves has not been investigated so far, and they are generally discarded, while stamens are enriched in polyphenols [16] and showed the highest antioxidant properties with respect to tepals [17]. Alongside studies on the spice saffron, it is important to assess the possible application of “waste” materials (tepals) as adjuvants in different diseases with consideration of a circular and sustainable economy.

The aim of the present review is to present the results of the flavonoids analysis in tepals obtained with different analytical techniques, providing a comprehensive collection and characterization of these important secondary metabolites. The literature data included in the present review cover the timespan 2003–2022, and data are presented according to the different classes of chemical compounds available in the floral tissues of *C. sativus*.

## 2. Phenolic Compounds

### 2.1. Flavonoids

The research interest in flavonoids derived from plants is due to their versatile health benefits reported in various epidemiological studies. Since flavonoids are directly associated with human dietary ingredients and health, there is a need to evaluate the structure/function relationship. The bioavailability, metabolism, and biological activity of flavonoids depend upon the nuclear structure configuration, the total number of hydroxyl groups, and the substitution of functional groups. Many flavonoids have been shown to exert different activities, such as antioxidant, free radical scavenging, hepato-protective, anti-inflammatory, and anticancer, while others exhibit potential antiviral activities and coronary heart disease prevention.

The biosynthesis of flavonoids has been extensively investigated, with nearly all the enzymes isolated and functionally characterized [18,19]. However, the pathways for sequential modification, such as glycosylation, acylation, and methylation, are still relatively unexplored, even though modification produces a large chemical diversity essential for the stable accumulation of flavonoids. Glycosylation induces more water solubility and less toxicity, and it may enable flavonol transport and compartmentation [20].

**Figure 2:**
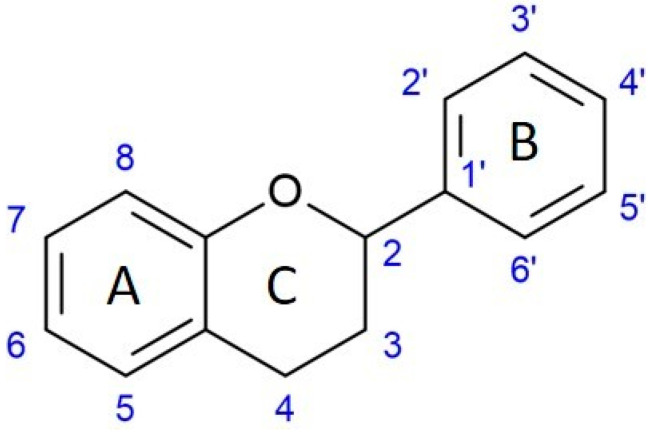
Structural architecture of flavonoids.

From the chemical point of view, flavonoids are made by two phenyl rings (A, B) and by a heterocycle pyrane ring (C) (Figure 2). According to the substitution and oxidation level of the C ring, different classes of flavonoids can exist, while the pattern of substitution at the A and B rings will determine different compounds within the classes.

Their variety within classes includes flavones (e.g., flavone, apigenin, and luteolin), flavonols (e.g., quercetin, kaempferol, myricetin, and fisetin), flavanones (e.g., flavanone, hesperetin, and naringenin), and others. Details on the chemical structures are summarized in Table S1. More details can be found in a recent review by Kumar and Pandey [21]. Flavonoids occur as aglycones, glycosides, and methylated derivatives. The majority of in vivo studies showed a good gastric absorption of aglycones such as quercetin and daidzein, while glycosides were reported to be poorly absorbed.

The total content of polyphenols, particularly flavonoids, may be related to the plant’s response to environmental conditions, such as light exposure [22,23]. In this respect, a recent study by Orlando [24] showed that floral by-products (tepals) from saffron plants grown under two LED treatments accumulated a higher amount of antioxidant compounds compared to those grown under natural light. The total flavonoid content was significantly enhanced by the RGB LED treatment, while the corolla fresh weight significantly declined in the same treatments.

Kaempferol 3-*O*-β-sophoroside is the main flavonoid in *C. sativus* L. flowers. This flavonol, extracted mainly from tepals, is about 100-times higher in content (g/(kg of dry weight) with respect to its presence in other foods considered “rich” in kaempferol. There are seven kaempferols glycosides, in addition to the aglycone, reported in saffron floral bioresidues. Moreover, in floral bioresidues, quercetin 3-*O*-sophoroside and isorhamnetin 3,4′-tri-*O*-glucoside, along with five anthocyanins, have also been reported. Kaempferols content, in decreasing order according to their concentration, are as follows: kaempferol 3-*O*-sophoroside, kaempferol aglycone, kaempferol 3-*O*-sophoroside-7-*O*-glucoside, kaempferol 3-*O*-rutinoside, kaempferol 3,7-di-*O*-glucoside, kaempferol 7-*O*-glucoside, kaempferol 3,7,40-tri-*O*-glucoside, and kaempferol 3-*O*-glucoside. With regards to anthocyanins, they include the following: delphinidin 3,5-di-*O*-glucoside, petunidin 3,5-di-*O*-glucoside, delphinidin 3-*O*-glucoside, malvidin 3,5-di-*O*-glucoside, and petunidin 3-*O*-glucoside. The analytical techniques usually adopted to investigate polyphenols and flavonoids are based on chromatographic methods, such as UPLC-DAD; hyphenated spectrometric methods, such as LC-DAD-MS/MS and LC-ESI-HRMS; and spectrophotometric methods, such as NMR. According to instrumental sensibility, different isomers can be detected.

Concerning the extraction procedures, different protocols have been explored to obtain the largest amount of bioactive components. Thus far, several phenolic compounds have been identified, such as benzoic acids, hydroxycinnamic acids [25], anthocyanins, and flavonols [26]. Additional procedures were proposed by Ahmadian-Kouchaksaraie using a supercritical carbon dioxide [27] and subcritical water extractions (SWE) [28]. In their studies, the dried and ground tepals from Iranian *C. sativus* L. were extracted with optimized conditions for both procedures (temperature of 62 °C, time of 47 min, and pressure of 164 bar for carbon dioxide, while the time of 54 min, temperature of 159 °C, and water/solid ratio of 36 mL/g were used for SWE). With the SWE approach, the dielectric constant of water can be decreased easily to that of ethanol or methanol by increasing the temperature. This condition favorably dissolves many compounds showing moderate or low polarity. Using carbon dioxide ensures it is non-toxic, non-flammable, available, cost-effective, easily removed from the extracted materials, well matched with the relatively low critical temperature (31.1 °C), and has a reduced thermal sample decomposition. The extracts from both procedures were checked for antioxidant activity and showed an increase in phenolic content.

In a recent study, Hashemi Gahruiea checked for bioactive compounds obtained from saffron tepals from Iran via three extraction approaches [29]. The authors compared extractions performed with hot water and three green innovative processing techniques, including ohmic-heating-assisted extraction, ultrasound-assisted extraction, and microwave-assisted extraction systems, considering the energy consumption, yield of bioactive compounds, and their physicochemical properties. The authors identified, using the LC-ESI-MS technique, the phenolic fraction obtained by the extraction approaches; the phenolic compounds were identified as gallic acid, catechin, vanillic acid, epicatechin, 3-hydroxyl-benzoic acid, isovanillin, rutin, naringin, benzoic acid, *orto*-cumaric acid, and quercetin. The identified flavonoids were delphinidin 3,5-di-*O*-glucoside, delphinidin 3-*O*-glucoside, petunidin 3,5-di-*O*-glucoside, petunidin 3-*O*-glucoside, and malvidin-*O*-glucoside. The optimized conditions for the extraction processes were sourced using a response surface methodology to identify the optimal power, voltage, and treatment time. Under the best conditions, the authors evaluated the highest content of bioactive compounds, based on their antioxidant activity, using the ohmic-heating-assisted extraction procedure.

In general, flavonols were mainly represented by kaempferol glycosides (84%), whereas quercetin and isorhamnetin glycosides represented only 9.3% and 8.7%, respectively [26,30,31,32,33].

There are few studies in which the bioavailability of different flavonols has been compared, and their results suggest that kaempferol is absorbed more efficiently than quercetin, while the latter is more extensively metabolized into other compounds [34,35,36].

A total of 21 different glycosides of kaempferol, quercetin, myricetin, naringenin, taxifolin, tamarixetin, and isorhamnetin have been identified in tepals [31]. With regards to flavonoid aglycones, kaempferol dominates, as kaempferol glycosides constitute between 70% and 90% of the total content of flavonoids in tepals; quercetin glycosides vary from 5% to 10%; and glycosides of dihydrokaempferol, isorhamnetin, naringenin, taxifolin, tamarixetin, and myricetin are present only as minor components [37].

A flavonoid fingerprint study performed using an LC-ESI-MS and LC-MS/MS approach was applied to saffron tepals by Montoro [33]. The methanolic extracts obtained with a 3-day extraction performed at room temperature revealed the presence of nine flavonoids, namely quercetin 3,7-di-*O*-β-d-glucopyranoside, kaempferol 3,7-di-*O*-β-d-glucopyranoside, isorhamnetin 3,7-di-*O*-β-d-glucopyranoside, kaempferol 3-*O*-β-d-glucopyranoside, quercetin 3-*O*-β-d-glucopyranoside, isorhamnetin 3-*O*-β-d-glucopyranoside, kaempferol 7-*O*-β-d-glucopyranoside, kaempferol 3-*O*-β-d-(2-*O*-β-d-glucosyl) glucopyranoside, and kaempferol 3-*O*-β-d-(2-*O*-β-d-6-*O*-acetylglucosyl) glucopyranoside, in addition to glycosidic derivatives of quercetin and kaempferol, as the major components.

Methanolic extracts from tepals of *C. sativus* L. from France were investigated by Goupy [38] using UPLC DAD/ESI-MS, and the flavonoid content was quantified by HPLC-DAD analysis at a 370 nm wavelength based on a calibration curve performed on kaempferol 3-*O*-glucoside. The analysis detected a main peak, with the addition of five other peaks and thirteen minor peaks. The major peak was due to kaempferol 3-*O*-glucoside, accounting for 55.4% of the total flavonol content. Other derivatives identified included kaempferol 3-*O*-sophoroside-7-*O*-glucoside and kaempferol-3-*O*-glucoside. In total, kaempferol glucosides accounted for 84% of the total flavonoid content. In addition, lutein di-esterified with lauric, palmitic, myristic, and stearic fatty acids were tentatively identified for the first time in *C. sativus* L. tepals.

Intact tepals of *C. sativus* L. from PDO production of Abruzzo (Italy) region were investigated by HR-MAS (high resolution magic angle spinning) NMR spectroscopy, by Righi [39]. The main advantage of the solid-state NMR analysis is the possibility to investigate intact solid materials without any sample preparation. In addition, alcoholic extraction of tepals was investigated by NMR in solution and LC-ESI/MS analysis to confirm the identification of three additional compounds, namely 3-(R)-3-β-d-glucopyranosyloxybutanolide (kinsenoside), 3-(S)-3-β-d-glucopyranosyloxybutanolide (goodyeroside), and 3-hydroxy-γ-butyrolactone, in combination with kaempferol 3-*O*-sophoroside.

Tepal extracts from PDO saffron have been purified by flash column chromatography and identified by thin layer chromatography (TLC), HPLC–DAD, infrared spectroscopy, and ^1^H and ^13^C NMR spectroscopy [40]. The amount of kaempferol was found to be relevant (126 mg/g of dry weight), particularly when compared with *Cruciferous* plants, e.g., broccoli, which are considered enriched in terms of kaempferol content, whose amount is only 1 mg/g of dry weight. The authors were looking for crocins and kaempferols; both these compounds are known to occur in *C. sativus* L. tepals, mainly as glycoside derivatives, e.g., seven different glycosides of kaempferol [26,38] and at least six different glycosides of crocetin [32]. The antioxidant activity was also determined with the ABTS and DPPH tests and confirmed the high amount of antioxidant compounds in saffron tepals.

Cusano and coworkers [41] investigated the content of bioactive compounds in different plant tissues of *C. sativus* L. cultivated in Italy using an integrated analytical approach. In particular, Raman, FT-IR and NMR spectroscopy, HPLC-DAD, and GC-MS spectrometry were applied to investigate the content of tepals, stamens, and stigma. Tepals revealed the presence of kaempferol 3,4′-di-*O*-glucoside as the most abundant compound, followed by kaempferol 3-*O*-β-sophoroside. In addition, kaempferol 3-*O*-sophoroside-7-*O*-glucoside and kaempferol 3,7′-di-*O*-glucoside were detected, together with isorhamnetin di-hexoside, in accordance with what was reported by many researchers as isorhamnetin 3,4′-di-*O*-glucoside.

Lahmass and coworkers [42] investigated the content of bioactive compounds in the waste of saffron crops, consisting of leaves, tepals, spathes, corm, and tunics. Saffron belonged to Oujda (Morocco), and the dried plant parts were extracted with methanol, ethyl acetate, and water/methanol 50:50 and investigated using LC-DAD-MS. The results showed that phenolic and flavonoid derivatives were present in higher amounts in stigmas, spathes, and leaves.

In a recent study, Xu [43] investigated different parts of saffron using a metabolomic approach using ultra-performance liquid chromatography coupled with high-definition mass spectrometry (UPLC-HDMS). The authors differentiated tepals, stigmas, and stamens based on specific metabolites; in particular, the content of astragalin, 1-monopalmitin, and kaempferol 3,7-di-*O*-β-d-glucoside were found to be remarkably higher in tepals than in the other parts of the plant.

Mottaghipisheh [44] recently investigated crocus flowers from 40 locations in Iran using HPLC-DAD analysis. The analysis of the methanolic extract of tepals showed kaempferol glycosides comprising kaempferol 3-*O*-sophoroside, kaempferol 3-glucoside, and kaempferol 3-*O*-d-(2-*O*-d-6-acetylglucosyl) glucopyranoside-7-*O*-d-glucopyranoside as the major compounds. Kaempferol 3-*O*-sophoroside was identified as the main component using HR-MAS combined with NMR spectroscopy. Other studies have also reported flavonoids from tepals, in particular kaempferol 3-*O*-sophoroside (K.S.), kaempferol 3-*O*-glucoside (K.G.), and quercetin 3-*O*-sophoroside (Q.S.). The results of a Mottaghipisheh study indicated flavonol glycosides K.S., K.G., and Q.S. as qualitative and quantitative marker compounds. In fact, their total amounts in dry tepals and stamen samples ranged between 62.19–99.48 mg/g and 0.90–6.62 mg/g for K.S., 27.74–45.18 mg/g and 1.72–7.44 mg/g for K.G., and 6.21–10.82 mg/g and 1.63–6.08 mg/g for Q.S., respectively. In general, K.S. was the main component in tepals (62.19–99.48 mg/g), while K.G. (1.72–7.44 mg/g) was the predominant constituent in stamens.

In a recent study [45], saffron cultivated in open fields in the northwestern Italian Alps was analyzed for 2 years. Interestingly, during this long experiment, plants produced more flowers per square meter and flowers per corm in the second cultivation cycle. It is well known that the flower yield is a difficult parameter to forecast in saffron cultivation, as it is influenced by a combination of agronomic, biological, and environmental factors. The authors adopted two extraction methods for fresh tepals: one based on ultrasound-assisted extraction for 15 min and the other based on maceration in the dark for 1 h. In addition, four different extraction solvents were tested: (a) deionized water; (b) deionized water:methanol (80:20 *v*/*v*); (c) deionized water:methanol (50:50 *v*/*v*); and (d) deionized water:methanol (20:80 *v*/*v*). The results are shown in Table 1. In particular, the 80% methanol extraction solution obtained a significantly higher quantity of phenolic compounds compared to the other solutions and more anthocyanins compared to the solution with 50% methanol. The bioactive compounds first identified in saffron tepals were determined to be hyperoside, rutin, ellagic acid, and epicatechin and were identified using high-performance liquid chromatography-diode array detection. This method involved employing various mobile phases to distinguish and analyze the compounds, while recording their UV spectra at different wavelengths.

The best extractions were performed with a methanol range from 50% to 80%, and in addition, ultrasonic-assisted extraction greatly reduced the composition differences between extracts with different percentages of extraction solvents. In greater detail, solvents with a low percentage of methanol (<20%) extracted a high amount of hyperoside, while a superior concentration ratio better extracted ellagic acid. A high content of methanol (>50%) was significantly effective for epicatechin.

Pappas [46] performed a comparative study of pressurized liquid extraction (PLE), stirred-tank extraction (STE), and stirred-tank extraction with ultrasonication pretreatment (STE/UP) on waste products (tepals) from Greek saffron. In an additional investigation, each extract was analyzed by LC/MS, which identified specific compounds. The most abundant flavonol glycoside was kaempferol 3-*O*-sophoroside, followed by kaempferol 3-*O*-sophoroside 7-*O*-glucoside, quercetin 3-*O*-sophoroside, and kaempferol 3-*O*-glucoside. This result was in accordance with recent findings on SPW extraction with a deep eutectic solvent [47]. Hierarchical cluster analysis indicated that stirred-tank extraction with 1% (*w*/*v*) lactic acid and ultrasonication pretreatment was the highest-performing combination, providing extracts with increased polyphenol and pigment concentration, which was also confirmed by enhanced antioxidant activity. Other identified compound anthocyanins were delphinidin 3-*O*-glucoside, followed by petunidin 3,5-di-*O*-glucoside and delphinidin 3,5-di-*O*-glucoside.

An interesting study was performed by Stelluti [48], evaluating the extraction protocols to optimize the amount of biomolecules obtained from dried saffron tepals. Conventional maceration was compared with ultrasound-assisted extraction (UAE) using different solvents (water and three different methanol concentrations, 20%, 50%, and 80%). The bioactive molecules detected were ferulic acid (cinnamic acid), ellagic acid, hyperoside, isoquercitrin, quercitrin, rutin, epicatechin (catechin), and vitamin C. The authors evaluated, as a general observation, the absence of the chemical compounds in fresh tepals; this effect might be attributed to the drying procedure, which can facilitate extractions by disrupting cell walls and causing the formation of cavities and intercellular spaces. A positive effect in gaining larger amounts of chemical compounds is observed when using UAE; this acoustic stimulation enables the permeability of the plant cell walls, improving solvent penetration and the release of bioactive compounds (Table 2). As a matter of fact, the authors evaluated a lower content of both total phenolics and anthocyanins with respect to the spice when a higher antioxidant activity was measured using FRAP, ABTS, and DPPH assays. In addition, HPLC-DAD analysis detected ferulic acid, isoquercitrin, and quercitrin not previously found in fresh saffron tepals. Vitamin C was also detected in dried tepals, and not only in the spice. Regarding the extraction technique, in most cases, UAE with safer solvents (i.e., water or a very low percentage of methanol) showed results of phenolic compounds and vitamin C similar to maceration, improving extractions by halving the time.

In a recent article, Gigliobianco [49] investigated the metabolite content of saffron tepals from two different Italian regions, Piemonte and Marche. Tepals were dried and the extraction procedure was performed using a microwave applicator endowed with selectable emitted power, time, and temperature in the presence of glycerine, water, or ethanol at 70 °C. Accurately weighed plant material (4.5 g) was added with solvent to a volume of 20 mL and then placed in a Pyrex™ vessel and heated under microwave irradiation (70 °C for 30 min). In their work, the authors identified 21 flavonols and anthocyanins, and the glycosylation position was determined for the first time in relation to the check of correct collision energy from 10 to 30 eV, useful for the cleavage of the glycosidic bond. It was possible to identify the di- and tri-glycosidic forms of kaempferol, a glycosylated species of isorhamnetin, while for delphinidin, the authors detected three signals at different collision energies based on the different positions of the glucosyl moiety, which could be in positions 3, 5, or 7 of the aglycone. In addition, two other flavonol species were detected and characterized by HRMS as a di-glucoside form of myricetin and primflasine, a glycosylated form of kaempferol. Concerning flavonols, the most abundant compound was found to be kaempferol 3-*O*-sophoroside, followed by quercetin 3-*O*-sophoroside, isorhamnetin 3-*O*-glucoside, and kaempferol 3,7,4′-*O*-triglucoside. The high content of kaempferol glycosides was in accordance with the results reported in the literature and detected in *C. sativus* L. tepals. In addition, five isomeric forms of glycoside myricetin and primflasine were detected.

Kaempferol was found to be stable over a wide range of different pH values, whereas quercetin requires an acidic pH to avoid oxidation [36].

In recent work, Ouahhoud [50] investigated the antioxidant capacity of bioactive compounds from different tepals, stigmas, and leaves of *C. sativus* L. from the Talouine region of Morocco. According to the extraction protocol reported in Table 3, the hydro-ethanolic extract from tepals revealed the largest amount of polyphenols (64.66 μg GA/mg extract) with respect to stigmas (34.41 μg GA/mg extract) and leaves (38.56 μg GA/mg extract). The flavonols were identified as kaempferol-3-*O*-sophoroside, quercetin, and isorhamnetin glucosides.

The antioxidant properties of tepals and stigmas were compared in a recent study by Bellachioma [51]. The metabolite profiles of the methanolic extracts from the two plant materials were investigated using UHPLC-QTOF spectrometry, revealing different phenolic compositions. In greater detail, phenolic profiling consisted of 452 compounds, namely, 66 anthocyanins, 17 flavan-3-ols, 67 flavonols, 94 other flavonoids (including flavones, flavanones, dihydrochalcones, dihydroflavonols, chalcones, and isoflavonoids), 29 lignans, 79 low-molecular-weight phenolics (i.e., tyrosol equivalents), 92 phenolic acids (mainly hydroxybenzoic and hydroxycinnamic acids), and 8 stilbenes. The total phenolic content differed between stigma and tepal extracts, 25.22 and 46.92 mg/g, respectively, and tepals were very abundant in flavonoids, with anthocyanins content of about 34.57 mg/g. In the latter, the most abundant compounds were identified as cyanidin, delphinidin 3-*O*-glucoside, malvidin 3-*O*-glucoside, pigment A (and its isomer peonidin 3-*O*-(6″-*p*-coumaroyl-glucoside)), petunidin 3,5-*O*-diglucoside, and malvidin 3,5-*O*-diglucoside. Moreover, terpenoids (e.g., carotenoids and xanthophylls) such as β-carotene, violaxanthin, and zeaxanthin have been detected in a larger amount in stigmas rather than in tepals. Multivariate statistical analysis of the two extracts showed clear discrimination based on their metabolite content. In this respect, the OPLS-DA score plot highlighted 38 compounds responsible for separating the two extracts. Among them, three flavonoids showed the highest variability, namely, 3-*O*-glucosyl-rhamnosyl-glucosides of kaempferol and quercetin, followed by the prodelphinidin trimer GC-GC-C. Other marker compounds exclusively characterizing tepal extracts were 1-sinapoyl-2,2′-diferuloylgentiobiose and 2,5-di-S-glutathionyl caftaric acid. Kakoury and coworkers [52] evaluated the hydrolyzed extract of crocus tepals, focusing on their phenolic composition, including flavonoids such as kaempherol and quercetin. These flavonoids are known for their potent antioxidant activity. The study found that crocus tepals exhibited significant antioxidant and antimicrobial activities. Regarding antimicrobial activity, crocus tepal extracts displayed antimicrobial efficacy against various strains of microorganisms. The hydrolyzed extract showed better antimicrobial activity compared to other extracts tested. The presence of kaempherol and quercetin in the phenolic composition of the tepals was identified as the key factor contributing to their antimicrobial capacity.

In recent years, “green” techniques have been developed to obtain natural extracts [53] and thus avoid the problems encountered when conventional methods are used (i.e., high energy and time consumption and the use of chemical solvents) [54]. Ultrasound-assisted (UA) extraction is considered an “environmentally friendly” or “green technique” compared to classical maceration [53,55] because it consumes less fossil energy, is more effective, and reduces the amount of solvent, resulting in higher yields with a shorter extraction time [54]. The selection of a suitable solvent is a crucial step in improving extraction efficiency; water and methanol are the most commonly used [56]. Nevertheless, water is only effective as an extraction solvent for polar compounds, while alcoholic solvents are efficient at extracting polar and weak polar compounds [57].

### 2.2. Anthocyanins

**Figure 3:**
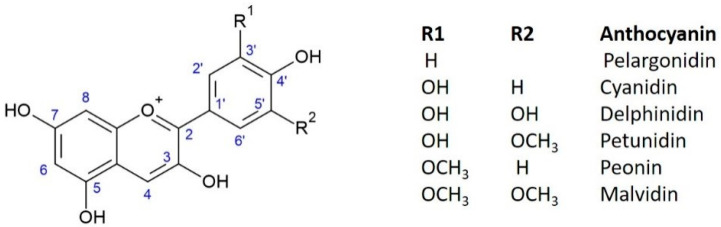
Schematic representation of anthocyanins backbone, with atom numbering.

Anthocyanins are ubiquitous water-soluble pigments responsible for the red, blue, and purple colors of plants and fruits. Generally, they are glycosylated derivatives of anthocyanidins, which, at a pH level below 2, appear in the form of flavylium cations. The structures of anthocyanins are characterized by the cyanidin aromatic ring and are categorized by the number of sugars and their position in the aglycon chain [58]. Anthocyanins are usually present as O-glycosides at the C3 position, although diglycosylation is also frequent, with di- or trisaccharide residues. They can also carry methoxylated residues, and the sugar moiety in C3 is commonly esterified with other aromatic acids (mainly hydroxycinnamic acids) or mono- and dicarboxylic aliphatic acids. The most common sugar is glucose, but arabinose, galactose, rhamnose, or xylose may also be present. On the other hand, the most common disaccharides include rutinose, lathyrose, sambubiose, and sophorose. In addition to the C3 position, other sugars can also be attached to any one of the hydroxyls [59,60,61]. There are six common anthocyanins, whose structures are shown in Figure 3.

The glycosidic units may be linked to the anthocyanidin by α or β linkage, and as already mentioned, is always in position 3 of the aglycon. When additional saccharides are present in the anthocyanin molecule, they are linked to positions 5 and 7, and, less frequently, to 3′ and 5′. Anthocyanins generally include two types: non-acylated and acylated. The saccharide present may be acylated; the most common aliphatic acylation is with acetic, malic, malonic, oxalic, succinic, and tartaric acids. On the other hand, aromatic acids include cinnamic, *p*-cinnamic, caffeic, gallic, and coumaric, as well as ferulic and sinapic. The acylated derivatives show more stability than their non-acylated equivalents. A recent study on tepals from *C. sativus* L. showed their abundance in flavonoids and anthocyanins. The colors of *C. sativus* L. flowers were reported to be predominantly based on delphinidin and petunidin presence with traces of malvidin derivatives. Anthocyanins are susceptible to light, temperature, and pH [62,63]. Several factors influence anthocyanin stability, including pH, oxygen, enzymes, ascorbic acid, sugars, sulfur dioxide or sulfite salts, and metal ions. Thus, many studies have reported strategies to increase the stability of these molecules [62,64,65,66]. Anthocyanins are soluble in polar solvents, and they are normally extracted from tepals using an acidic solution of methanol (containing small amounts of hydrochloric acid or formic acid); they are relatively unstable and easily subjected to oxidation.

Moratalla-López and coworkers [67] investigated the total anthocyanins content in saffron floral bio-residues and whole flowers during storage and after dehydration. The authors demonstrated that temperature and relative humidity affected the stability of dry saffron floral bio-residues and whole flowers. The most convenient storage conditions to avoid a loss of anthocyanins were established as 25 °C and 23% relative humidity. On the contrary, 40 °C and 75% relative humidity produced the fastest degradation of anthocyanins.

Lotfi and coworkers [68] studied the effects of a SO_2_ aqueous solution on the pigment of saffron tepals by measuring the quantity and quality criteria of its extracted anthocyanins. The extraction of anthocyanins was carried out using an aqueous solution of sodium metabisulfite. The anthocyanin content was significantly higher than when extracted with an acidified ethanol solution, and, in addition, a sulfur derivate was able to stabilize monomeric and polymeric forms of anthocyanins. In addition, Lofti reported using suitable food-grade enzymes for the aqueous recovery of anthocyanins from saffron tepals, whose yield, quality, and properties were compared with those extracted using the conventional ethanol method. The enzymes used provided a significantly higher efficiency in extracting anthocyanins from saffron tepals with respect to the extraction with the alcoholic solution. Furthermore, the enzyme-extracted anthocyanins showed resistance against browning and decomposition processes and higher stability than those obtained from the acidified ethanol. In a recent work, Lotfi [69] used different concentrations of metabisulfite in the sulfur solution and various extraction times at 40 °C. They found that the recovery of anthocyanins with the sulfur solution was higher compared to ethanol extraction. The optimal conditions for sulfur extraction were a concentration of 700 ppm and an extraction time of 60 min, resulting in the highest recovery of 700 mg anthocyanins per 100 g of saffron tepals. High-performance liquid chromatography analysis revealed that anthocyanins extracted with sulfur solution, followed by partial desulfurization to reduce sulfur content, had significantly higher levels of cyanidin 3 glucosides and lower levels of pelargonidin 3,5 glucosides compared to ethanol extraction. The color of the extracted anthocyanins using the sulfur method also exhibited more saturation, less lightness, and better stability than those extracted with ethanol solution. Furthermore, the sulfur extraction method showed less than 1% change in monomeric and polymeric anthocyanins after 3 h of extraction, while extraction with ethanol resulted in more than 12% changes under similar conditions. Overall, the sulfur method demonstrated the potential to extract stable anthocyanins from saffron tepals, which are typically discarded as waste. The extracted anthocyanins had higher quantity and quality, including more attractive color properties, compared to the conventional ethanol extraction method. The study provides valuable insights into an alternative extraction method for natural pigments and highlights the potential of utilizing waste saffron tepals for the production of stable anthocyanins in aqueous solvents.

Cerdà and coworkers [70] explored the use of microwave-assisted extraction (MAE) to obtain high-value compounds from saffron floral by-products. The study aimed to optimize the MAE process variables, including temperature, time, and ethanol solvent concentration, using response surface methodology. The researchers conducted a central composite design experiment to evaluate the effects of the process variables on the extraction yield, total phenolic and flavonoid content, and antioxidant capacity of saffron floral extracts. The results showed that the optimal extraction conditions were a combination of low temperature (25 °C), high extraction time (5 min), and the use of ethanol as a solvent. Under these conditions, the extracts exhibited maximum values of 126.20 ± 2.99 mg gallic acid equivalent (GAE)/g dry matter for total phenolic content, 8.05 ± 0.11 mg catechin equivalent (CE)/g dry matter for total flavonoid content, 6219 ± 246 μmol trolox equivalent antioxidant capacity (TEAC)/g dry matter (measured by oxygen radical absorbance capacity), and 3131 ± 205 μmol TEAC/g dry matter (measured by hydroxyl radical scavenging capacity). The study demonstrated that MAE is an efficient technique for isolating bioactive compounds from saffron floral by-products with a low energy footprint. The optimized extraction process using MAE can potentially lead to the development of high-value ingredients for use in the food, pharmaceutical, and cosmetic industries. An innovative extraction method was developed by Vardakas and coworkers [71]. The study utilizes a combination of subcritical water extraction (SWE) and enzymatic treatment to recover polyphenols from saffron tepals. The process involves two SWE steps with an enzymatic treatment incorporated between them. Liquid chromatography mass spectrometry was used to characterize the polyphenol profile of the extract. Additionally, the co-pigmentation efficiency of the polyphenol extract and its impact on the thermal stability of strawberry anthocyanins were evaluated in model solutions. The results showed that the combined extraction method led to a four-fold increase in the polyphenolic yield compared to conventional methods. The saffron tepal extract contains 16 flavonoids, primarily flavonols. The presence of the flavone apigenin in *C. sativus* was reported for the first time.

Moratalla-Lopez [67] reported the anthocyanins in decreasing order according to their concentration in tepals as follows: delphinidin 3,5-di-*O*-β-glucoside, petunidin 3,5-di-*O*-β-glucoside, delphinidin 3-*O*-β-glucoside, malvidin 3,5-di-*O*-β-glucoside, and petunidin 3-*O*-β-glucoside. In recent studies, Serrano-Díaz [30] evaluated several solvents to extract freeze-dried saffron floral to obtain anthocyanins. The authors adopted six solvents: deionized water, a mixture of water/HCl (100:1, *v*/*v*), ethanol, a mixture of ethanol/HCl (100:1, *v*/*v*), dichloromethane, and hexane. The six extracts were stirred, centrifugated, and then concentrated under a vacuum to identify and quantify anthocyanins using HPLC-ESI-DAD-MS^n^. The highest total anthocyanins concentration was observed in the non-acidified aqueous extract in the amount of 24.91 mg/g, expressed as mg of delphinidin 3,5-diglucoside equivalents/g dried weight. The mixture of water/HCl provided 14.92 mg/g, while the rest of the extracts showed much lower values, ranging between 0.27 and 3.67 mg/g. Delphinidin 3,5-diglucoside, followed by petunidin 3,5-diglucosideC and followed by delphinidin 3-glucosideC, were the major anthocyanins in water. Delphinidin 3,5-diglucoside has been described as the main anthocyanin [37]. However, in a mixture of water/HCl, the largest found was delphinidin 3-glucoside C. This could be due to the acidic conditions that hydrolyzed the glycosidic bond of delphinidin 3,5-diglucoside. Goupy and coworkers [38] identified the major anthocyanins of *C. sativus* L. tepals using ultra-performance liquid chromatography with a diode array detection coupled to an ion trap mass spectrometer with electrospray or atmospheric pressure chemical ionization. The extraction procedure was carried out at room temperature. The powdered dried tepals were extracted by ultrasonic treatment for 5 min in 20 mL methanol/water (50:50, *v*/*v*) containing HCl 1%, followed by magnetic stirring. The supernatants were collected and concentrated in a rotary evaporator under reduced pressure at 25 °C. The residue was thus dissolved in methanol containing 1% HCl, filtered, and stored in an amber vial at −20 °C. For qualitative studies, extracts were analyzed by ultra-performance liquid chromatography (UPLC) linked to both a diode array detector 190–800 nm and an Ultra Ion Trap MS equipped with an electrospray ion source or an atmospheric pressure chemical ionization source. Anthocyanins analysis was carried out using a BEH C_18_ column and the temperature was set at 35 °C. The analysis was achieved with a gradient elution using water containing 0.1% HCl (solvent A) and acetonitrile (solvent B) as the mobile phase. The peaks of anthocyanins were monitored at 530 nm. For the UPLC DAD/ESI-MS^n^ and UPLC DAD/APCI-MS^n^ analyses, the injection volume was fixed at 1 µL. The ion trap was set in the ultra-scan mode from *m*/*z* 120 to 1400. Ionization was achieved using an ESI source in positive mode to detect anthocyanins. All anthocyanins’ UV–vis spectra in acidic methanolic solution showed two separate bands: one in the UV region (270–276 nm) and another in the visible region (521–525 nm). The content of anthocyanins in methanolic extracts was quantified by UPLC DAD analysis with a detection wavelength at 530 nm against a calibration curve obtained from the dilution series of delphinidin 3-*O*-glucoside. The ESI^+^−MS^n^ analysis detected five different [M]^+^ molecular ions. MS and MS^2^ spectra of anthocyanins peaks displayed molecular ions characteristic of delphinidin, petunidin, or malvidin glucosides with an intense signal for the flavylium cation.

Serrano-Díaz and coworkers [30] proposed a validated chromatographic method, coupled with electrospray ionization mass spectrometry (ESI-MS^n^), for the analysis of anthocyanins in saffron tepals using three different solvent mixtures: water/ hydrochloric acid (HCl) (100:1, *v*/*v*), water/acetonitrile/trifluoroacetic acid (47:50:3, *v*/*v*/*v*), and water/acetonitrile/HCl (50:50:1, *v*/*v*/*v*). The extracts showed various phenolic profiles. The results obtained in this study showed that the extract prepared with water/HCl (100:1, *v*/*v*) was the best suited for determining anthocyanins in tepals of *C. sativus* L.

Recently, Nørbæk and coworkers [37] presented a chemotaxonomic study by determining anthocyanins and other flavonoids using analytical high-performance liquid chromatography (HPLC). This study was performed on 70 species and subspecies, 43 cultivars, and 6 artificial hybrids of *Crocus.* Nine anthocyanins were identified by modern NMR techniques as 3,7-di-*O*-β-glucosides, 3,5-di-*O*-β-glucosides, and 3-*O*-β-rutinosides of delphinidin and petunidin, respectively, and delphinidin 3-*O*-β-glucoside-5-*O*-(6-*O*-malonyl-β-glucoside) and 3-*O*-(6-*O*-malonyl-β-glucoside)-7-*O*-(6-*O*-malonyl-β-glucoside) of petunidin and malvidin. Recently, Nørbæk and Kondo [72] isolated new anthocyanins via column chromatography on Amberlite XAD-7 with subsequent preparative HPLC. Fast atom bombardment mass spectroscopy (FAB-MS) was used to identify the anthocyanins. For example, delphinidin 3-glucoside-5-(6-malonyl) glucoside was identified on the basis of a molecular peak [M]^+^ at *m*/*z* 713, in good agreement with the mass calculated for C_30_O_20_H_33_^+^. The fragment peaks were observed at *m*/*z* 551 [M-162 (hexose)]^+^, 465 [M-248 (malonyl-hexose)]^+^, and 303 [aglycone]^+^. The structure was confirmed by NMR analysis. The ^1^H NMR spectrum revealed the presence of two glucose residues, one acylated with malonic acid. The assignments of the two hexoses were completely carried out by one-dimensional homonuclear Hartmann–Hahn spectroscopy (HOHAHA) spectra and correlation spectroscopy (COSY). The positions of the glucosidic linkages were determined by nuclear Overhauser effect (NOE) difference spectra.

Table 4 summarizes the collected references for the extraction methods and the identification of the main components in *C. sativus* L. tepals.

The scientific community considers anthocyanins as promising substances with significant nutraceutical applications [73,74], and many studies have demonstrated the antimicrobial activities of these compounds [75,76,77]. These activities are due to the ability of anthocyanins to destroy the cell wall and cell membrane of Gram-negative bacteria. Moreover, some anthocyanins can interfere directly with the metabolism of some microbes, depriving them of fundamental substrates for their growth [75].

The literature on anthocyanin anti-tumor activity is also abundant. These molecules (such as cyanidine 3-glucoside, C3G) are able to limit the carcinogenic activity of ethanol, preventing metastasis [78]. The anticancer capacity of anthocyanins is linked to their tendency to induce apoptosis in cancer cells and suppress angiogenesis [79].

In addition to anti-tumor activity, anthocyanins have also shown antidiabetic activities, particularly against diabetes mellitus [80]; they can prevent a rise in blood glucose levels and improve insulin resistance [81,82].

With an aging population, concerns about neurodegenerative diseases are becoming increasingly relevant topics. Multiple pathways, including apoptosis, autophagy, mitochondrial dysfunction, and oxidative DNA damage and repair, have been identified in different neurodegenerative diseases; however, the functional mechanistic context in each disease is different. In fact, the mechanisms of neurodegenerative diseases are still far from being clarified, which is a major challenge for the discovery of a potential therapy that can help to delay the effects of aging and prevent these diseases.

Saffron is known to have neuroprotective activity, as demonstrated by several scientific productions [83,84]. Among the main components of saffron tepal extracts, anthocyanins have now become a topic of interest as a natural preventive/therapeutic strategy because they have the ability to protect neurons against oxidative stress, suppress neuroinflammation, and modulate cell-signaling pathways.

In summary, animal studies and random clinical trials suggest that anthocyanins improve cognition and neuroprotection. According to in vivo studies, the mechanisms responsible for these benefits are related to anthocyanins’ ability to decrease oxidative stress, inflammation, and degeneration in the brain. Further research must focus on finding the dose and the frequency of the treatment with anthocyanins that can be applied to humans to attain neuroprotective benefits [85,86,87,88,89,90,91,92].

## 3. Conclusions

In conclusion, the present review focused on the comprehensive analysis and characterization of secondary metabolites in the tepals of *C. sativus* L. This autumnal herbaceous flowering plant is primarily cultivated for its valuable spice, saffron, which is derived from the stigmas. However, the tepals, which are considered by-products, contain a significant amount of plant material that remains largely unused. Tepals have been found to be a potential source of bioactive compounds with various health benefits. The analysis of tepals has revealed the presence of a wide range of secondary metabolites, particularly phenolic compounds such as flavonoids. Flavonoids have been extensively studied due to their versatile health benefits, including antioxidant, anti-inflammatory, and anticancer properties. The flavonoids in tepals include different classes such as flavones, flavonols, and flavanones, with kaempferol glycosides being the most abundant. Several studies have investigated different extraction techniques to obtain a higher yield of bioactive compounds from tepals. Optimized extraction methods using solvents such as water, methanol, or ethanol have been shown to enhance the extraction efficiency and increase the content of phenolic compounds. Additionally, innovative techniques such as ultrasound-assisted extraction and microwave-assisted extraction have been explored, providing promising results in terms of improving the extraction efficiency and preserving the bioactive compounds. The findings of the reviewed studies highlight the potential use of tepals as a valuable resource for the development of functional foods, nutraceuticals, and pharmaceutical products. The abundance of bioactive compounds, particularly flavonoids in tepals, suggests their potential application in various industries, including the food, cosmetic, and pharmaceutical sectors. Furthermore, the utilization of tepals as animal feed or fertilizer could contribute to a circular and sustainable economy. Future research should focus on further understanding the biosynthetic pathways and modification processes of flavonoids in tepals. Additionally, exploring the bioavailability, metabolism, and specific health benefits of individual flavonoid compounds would provide valuable insights into their potential therapeutic applications. Overall, the analysis of secondary metabolites in tepals has provided valuable information about their chemical composition and potential health benefits. Further exploration and utilization of these bioactive compounds could contribute to the development of novel products and promote sustainable and circular approaches in the saffron industry.

## Figures and Tables

**Table 1:** Total polyphenol content (TPC, mgGAE 100 g^−1^ FW), anthocyanins (mgC3G 100 g^−1^ FW), antioxidant activity (FRAP, mmol Fe^2+^ kg^−1^; DPPH, µmolTE g^−1^; ABTS, µmolTE g^−1^ assays), and bioactive compounds (mg 100 g^−1^ FW) in fresh saffron tepal extracts obtained with maceration (M) and ultrasound-assisted (UA) extraction, varying percentage of methanol in aqueous solvents (0%, 20%, 50%, and 80%). Data are presented as the mean ± standard deviation. Reproduced from Ref. [45].

			Antioxidant Activity			Flavonols		Benzoic Acids	Catechins
Extraction Method	TPC	Anthocyanins	FRAP	DPPH	ABTS	Hyperoside	Rutin	Ellagic Acid	Epicatechin
M	465.7 ± 36.4	119.7 ± 15.6	167.3 ± 10.2	5.53 ± 0.79	5.84 ± 1.1	2.00 ± 0.72	0.15 ± 0.04	1.63 ± 0.35	3.13 ± 0.72
UA	486.9 ± 41.8	119.3 ± 12.8	141.1 ± 7.3	6.06 ± 1.12	8.88 ± 0.97	2.42 ± 0.37	0.13 ± 0.05	3.06 ± 0.66	5.27 ± 0.42
*p*	ns	ns	**	ns	***	ns	ns	***	*
**Solvent (% methanol)**									
0%	449.8 ± 35.5 ^b^	142.5 ± 23.5 ^a,b^	253.5 ± 5.3 ^a^	4.61 ± 1.27	5.56 ± 1.03 ^b^	2.96 ± 0.64 ^a^	0.16 ± 0.07	1.42 ± 0.35 ^b^	0.00 ± 0.00 ^b^
20%	374.8 ± 47.2 ^b^	103.3 ± 18.9 ^a,b^	108.8 ± 11.7 ^c^	4.82 ± 1.86	6.45 ± 0.98 ^b^	2.98 ± 0.59 ^a^	0.09 ± 0.02	2.52 ± 0.18 ^a^	0.00 ± 0.00 ^b^
50%	402.9 ± 28.0 ^b^	88.8 ± 15.9 ^b^	105.9 ± 15.2 ^c^	6.99 ± 1.43	10.53 ± 1.23 ^a^	1.34 ± 0.79 ^b^	0.16 ± 0.10	2.71 ± 0.31 ^a^	9.18 ± 0.97 ^a^
80%	677.7 ± 23.4 ^a^	143.3 ± 22.3 ^a^	148.6 ± 10.4 ^b^	6.77 ± 1.76	6.79 ± 0.72 ^b^	1.53 ± 0.68 ^b^	0.21 ± 0.09	2.72 ± 0.22 ^a^	7.62 ± 1.65 ^a^
*p*	***	*	***	ns	***	***	ns	**	***
**Interaction**									
**Extraction × Solvent**	ns	ns	**	ns	***	***	ns	ns	*

Same letter denotes no significant differences according to Tukey post-hoc test (*p* < 0.05). The statistical relevance is provided (ns = non-significant; ** = *p* < 0.01; *** = *p* < 0.001). The statistical relevance of “between-subjects effects” tests is provided (ns = non-significant; * *p* < 0.05; ** *p* < 0.01; *** = *p* < 0.001).

**Table 2:** Extraction yield (mg 100 g^−1^ DW) of the compounds obtained from dried saffron tepals expressed as mg 100 g^−1^ of dried weight (DW) using maceration (M) and ultrasound-assisted extraction (UAE) techniques, as well as the solvents water or methanol at three concentrations (20%, Met20; 50%, Met50; 80%, Met80; *v*:*v*). Quantifications were obtained by HPLC-DAD analysis. (Reproduced from Ref. [48]).

Extractions		Cinnamic Acids	Benzoic Acids	Flavonols				Catechins	Vitamin C
		Ferulic Acid	Ellagic Acid	Hyperoside	Isoquercitrin	Quercitrin	Rutin	Epicatechin	
M	Water	0.00 ± 0.00 b	7.67 ± 3.69 a,b,c	4.35 ± 1.04 c	0.31 ± 0.22 c,d	0.00 ± 0.00 b	8.52 ± 3.91 c	0.00 ± 0.00 b	29.61 ± 6.05 a
M	Met20	1.83 ± 0.31 a	4.43 ± 4.15 c,d	5.61 ± 0.52 a,b,c	0.22 ± 0.12 c,d	6.33 ± 5.27 a	0.32 ± 0.31 d	0.00 ± 0.00 b	33.72 ± 0.89 a
M	Met50	9.65 ± 2.62 a	0.00 ± 0.00 e	5.85 ± 4.31 b,c	4.36 ± 3.49 a,b,c	9.27 ± 3.47 a	0.00 ± 0.00 d	0.00 ± 0.00 b	0.00 ± 0.00 b
M	Met80	0.00 ± 0.00 b	1.32 ± 0.33 d,e	23.93 ± 15.51 a,b,c	7.82 ± 3.09 a	6.53 ± 0.29 a	37.61 ± 2.22 a	0.00 ± 0.00 b	0.00 ± 0.00 b
UAE	Water	0.00 ± 0.00 b	8.53 ± 8.45 b,c,d	11.58 ± 4.09 a,b,c	0.00 ± 0.00 d	0.00 ± 0.00 b	28.24 ± 4.83 a,b	0.00 ± 0.00 b	26.68 ± 4.71 a
UAE	Met20	0.00 ± 0.00 b	26.74 ± 10.80 a,b	9.68 ± 6.77 a,b,c	6.46 ± 5.03 a,b	0.00 ± 0.00 b	13.46 ± 10.25 b,c	16.62 ± 15.89 a	29.17 ± 2.31 a
UAE	Met50	0.00 ± 0.00 b	28.39 ± 4.32 a	27.26 ± 4.29 a	5.57 ± 1.90 a,b,c	7.07 ± 5.12 a	7.24 ± 1.35 c	0.00 ± 0.00 b	0.00 ± 0.00 b
UAE	Met80	0.00 ± 0.00 b	23.51 ± 5.11 a,b	24.77 ± 2.25 a,b	0.00 ± 0.00 d	0.00 ± 0.00 b	9.10 ± 2.17 c	4.22 ± 2.90 a	0.00 ± 0.00 b
*p*		0.001802 **	2.235 × 10^−7^ ***	0.004662 **	0.005466 **	0.005407 **	1.452 × 10^−10^ ***	0.001995 **	0.003143 **

Values of mean ± standard deviation are reported. Statistical comparisons were performed using ANOVA (for ellagic acid, hyperoside, and rutin) or Kruskal–Wallis test (for the other compounds, *p* < 0.05 in Shapiro–Wilk’s test). Letters indicate statistical differences between the different extractions for each extracted compound. Values with the same letter are not statistically different at *p* < 0.05, according to Tukey’s or Dunn’s post-hoc test. ** *p* < 0.01; *** *p* < 0.001; ns = not significant.

**Table 3:** Total polyphenol content of the different hydroethanolic extracts from the stigmas, tepals, and leaves of *C. sativus* L. Reproduced from Ref. [50].

Sample	Polyphenol Content (µg GA eq/mg Extract)
STG	(34.41 ± 1.09)
TPL	(64.66 ± 0.20)
LV	(38.56 ± 0.34)

Values are expressed as mean ± SEM (*n* = 3). GA eq: gallic acid equivalent; STG = hydroethanolic extract of stigmas; TPL = hydroethanolic extract of tepals; LV = hydroethanolic extract of leaves.

**Table 4:** Collected references for the different extraction methods and the identification of the main components in *C. sativus* L. tepals.

**Extraction Method**	**Pre-Status**	**Main Compounds**	**Reference**	**Ref. N°.**
acidified methanol 80%, sonicated for 30 min in ice bath	frozen	flavonol glycosides and anthocianis	Orlando et al., 2022	[24]
Subcritical water	dried	phenolic compounds	Ahmadian-Kouchaksaraie et al., 2016	[27]
Supercritical Carbon Dioxide	dried	phenolic compounds, anthocyanins, flavonoids	Ahmadian-Kouchaksaraie et al., 2017	[28]
(a) hot water, (b) ohmic heating assisted extraction, (c) ultrasound assisted extraction, (d) microwave assisted extraction	dried	kaempferol derivatives and anthocyanins	Hashemi Gahruiea et al., 2020	[29]
(a) water/HCl 100:1 at 40 °C by stirring for 1h. (b) water/ACN/TFA (47:50:3). (c) water/ACN/HCl (50:50:1). (d) water/ethanol/HCl (50:50:1). (e) water/acetone/HCl (50:50:1)	frozen	kaempferol 3-*O*-sophoroside, kaempferol 3-*O*-glucoside, kaempferol, delphinidin 3,5-*O*-diglucoside, petunidin 3,5-*O*-diglucoside	Serrano-Diaz et al., 2014	[30]
(a) diethyl ether, (b) ethyl acetate, (c) aqueous	-	kaempferol, quercetin, naringenin, flavanone and flavanol derivatives glycosylated and esterified with phenylpropanoic acids.	Termentzi et al., 2008	[31]
methanol	-	flavonoids, glycosidic derivatives of quercetin and kaempferol	Montoro et al., 2008	[33]
dichloromethane, methanol, acetonitrile, diethyl ether, n-hexane and ethyl acetate	vacuum freeze drying	glycosilated forms of kaempferol, isorhamnetin, quercetin, glycolilated forms of anthocyanins	Goupy et al., 2013	[38]
ethanol	fresh	kinsenoside, goodyeroside, 3-hydroxy-γ-butyrolactone, kaempferol 3-*O*-sophoroside	Righi et al., 2015	[39]
methanol/HCL 9:1	dried	kaempferol 3-*O*-sophoroside-7-*O*-glucoside, quercetin 3,4′-di-*O*-glucoside, delphinidin 3,5-di-*O*-β-glucoside, petunidin 3,5-di-*O*-β-glucoside, delphinidin 3-*O*-β-glucoside, petunidin 3-*O*-β-glucoside kaempferol 3,7′-di-*O*-glucoside,	Cusano et al., 2018	[41]
methanol/water 1:1 with ultrasonication	dried	astragalin, 1-monopalmitin, kaempferol-3,7-di-*O*-β-d-glucoside	Xu et al., 2019	[43]
ethanol/water 1:1 with ultrasonication	dired	kaempferol-3-*O*-sophoroside, quercetin-3-*O*-sophoroside, kaempferol-3-*O*-glucoside;	Mottaghipisheh et al., 2020	[44]
deionised water; deionized water:methanol (80:20 *v/v*); deionised water:methanol (50:50 *v/v*); deionised water:methanol (20:80 *v/v*). assisted extraction	fresh	hyperoside, rutin, ellagic acid, epicatechin, flavonols	Caser et al., 2020	[45]
Pressurized-Liquid Extraction	freeze dried	kaempferol 3-*O*-sophoroside, kaempferol 3-*O*-sophoroside 7-*O*-glucoside, quercetin 3-*O*-sophoroside, kaempferol 3-*O*-glucoside,. Delphinidin 3,5-di-*O*-glucoside, delphinidin 3-*O*-glucoside, petunidin 3,5-di-*O*-glucoside, delphinidin 3,5-di-*O*-glucoside	Pappas et al., 2021	[46]
Deep eutectic solvent (lactate and glycine)	dried	kaempferol 3-*O*-sophoroside 7-*O*-glucoside, quercetin 3-*O*-sophoroside, kaepferol 3-*O*-sophoroside, kaempferol 3-*O*-glucoside, delphinidin 3,5-di-*O*-glucoside, petunidin 3,5-di-*O*-glucoside, delphinidin 3-*O*-glucoside	Lakka et al., 2009	[47]
Ultrasound Assisted Extraction, using different water and methanol ratios like 20%, 50%, and 80%		ferulic acid (cinnamic acid); ellagic acid; hyperoside, isoquercitrin, quercitrin, rutin, epicatechin (catechin), vitamin C	Stelluti et al., 2021	[48]
ultrasound assisted extractio of 4.5 g with 20 mL ethanol at 70 °C for 20 min	dried	kaempferol 3-*O*-glucoside, isorhamnetin 3-*O*-glucoside, kaempferol 3,7,4′-*O*-triglucoside, delphinidin 3,5-di-*O*-glucoside, myricetin-di-glucoside, primflasine	Gigliobianco et al., 2021	[49]
80/20 ethanol/water for 24 H	dried	kaempferol-3-*O*-sophoroside, quercetin and isorhamnetin glucosides.	Ouahhoud et al., 2022	[50]
methanol/MTBE 1:1	freeze dried	several compounds divided into chemical classes	Bellachioma et al., 2022	[51]
water/HCl (100:1 *v/v*)	dried	delphinidin 3,5-di-*O*-β-glucoside, petunidin 3,5-di-*O*-β-glucoside, delphinidin 3-*O*-β-glucoside, malvidin 3,5-di-*O*-β-glucoside and petunidin 3-*O*-β-glucoside	Moratalla-López et al., 2017	[67]
ethanol/HCl (85:15 *v/v*)	freeze dried	Pelargonidin 3-glycosides,Pelargonidin 3,5-glycosides, Petunidin, 3,5 Cyanidin-diglycosides, Delphinidin 3-glycosides	Lofti et al., 2015	[68]
1 g extracted with 13 mL acetonitrile/water 1:1 containing 3.0% trifluoroacetic acid	freeze dried	several compounds divided into chemical classes	Nørbæk 2002	[37]
50 g extracted with acetonitrile/water 1:1 containing 0.5% trifluoroacetic acid	freeze dried	delphinidin 3-glucoside-5-(6-malonyl)glucoside, delphinidin 3,7-diglucosides, petunidin 3,7-diglucosides, delphinidin 3,5-diglucosides, petunidin 3,5-diglucosides	Nørbæk 1999	[72]

## Data Availability

Not applicable.

## Supplementary Materials

The following supporting information can be downloaded at: https://www.mdpi.com/article/10.3390/molecules28165976/s1, Table S1: Detailed chemical structures of the main secondary metabolites belonging to flavonoids and anthocyanins classes.
