# Supplementary material for: Comprehensive Extraction and Chemical Characterization of Bioactive Compounds in Tepals of Crocus sativus L
Source: Molecules. 2023 Aug 9;28(16):5976. doi: 10.3390/molecules28165976 (PMC10458886; doi:10.3390/molecules28165976)
Supplement: Supplementary file 1 [file molecules-28-05976-s001.zip › molecules-2420413-supplementary.pdf]

Comprehensive Extraction and Chemical Characterization of Bioactive Compounds in Tepals of *Crocus Sativus* L.

Fabrizio Ruggieri [1], Maria Anna Maggi [1], Michela Rossi [1] and Roberto Consonni [2,*]

supplementary materials

Table S1: Detailed chemical structures of the main secondary metabolites belonging to flavonoids and anthocyanins classes.

kaempferol	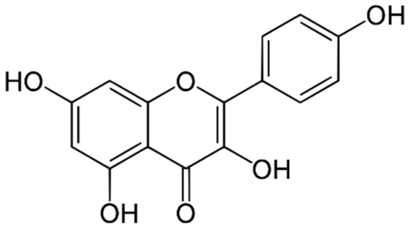
kaempferol 3-*O*-β-sophoroside	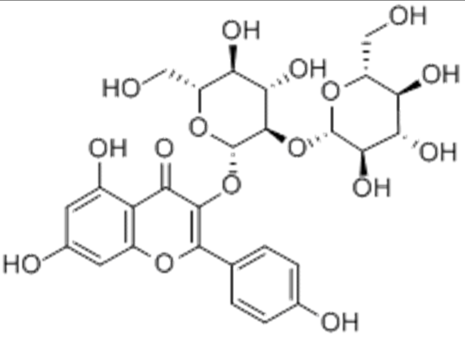
kaempferol 3,4-di-*O*-glucoside	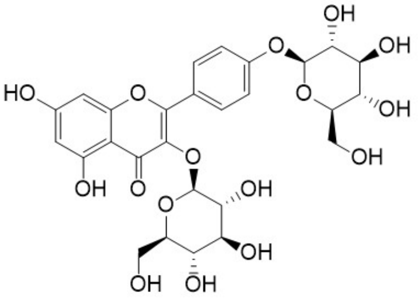
kaempferol 3-*O*-rutinoside	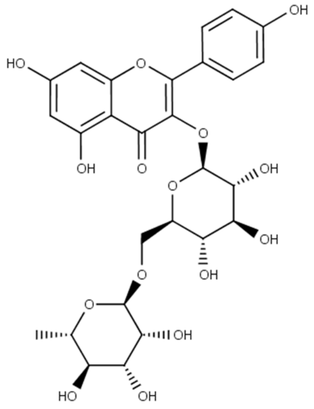

kaempferol 3,7-di-*O*-glucoside	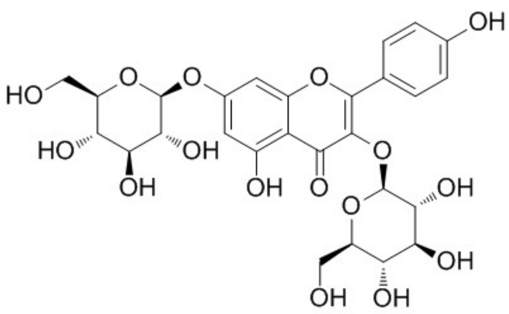
kaempferol 7-*O*-glucoside	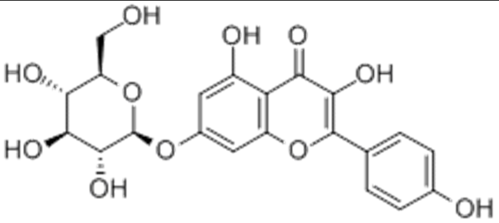
kaempferol 3,7,4′-tri-*O*-glucoside	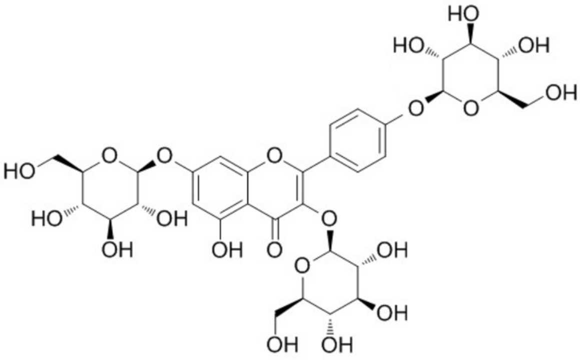
kaempferol 3-*O*-glucoside	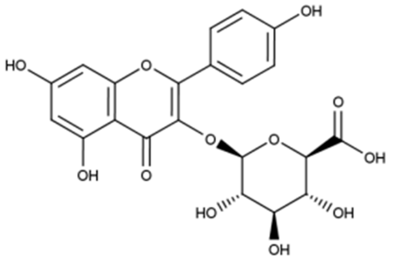
kaempferol 3,7-di-*O*-β-D-glucopyranoside	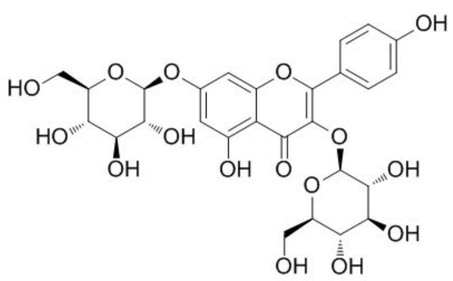
kaempferol 7-*O*-β-D-glucopyranoside	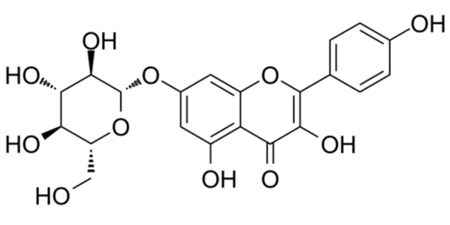

kaempferol 3-*O*-sophoroside-7-*O*-glucoside	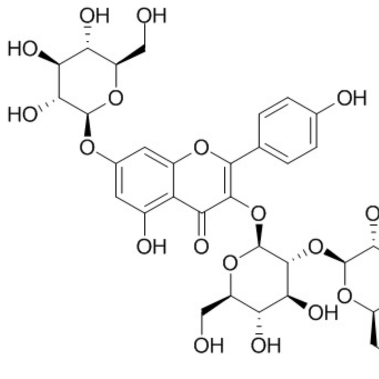
quercetin	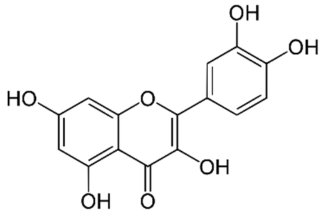
quercitrin	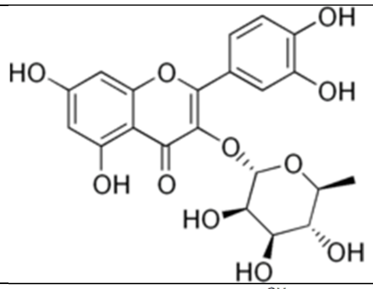
quercetin 3-*O*-sophoroside	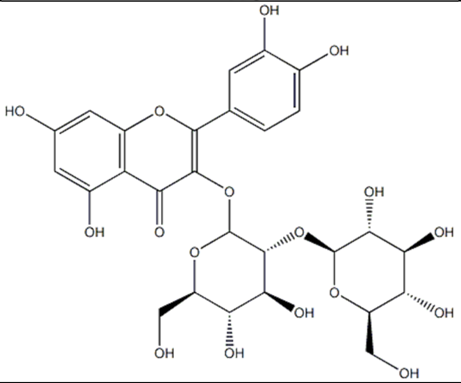
quercetin 3-*O*-β-D-glucopyranoside	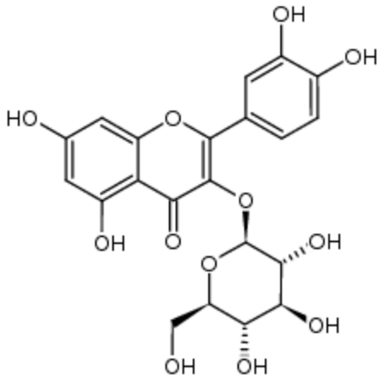

quercetin 3,4′-di-*O*-glucoside	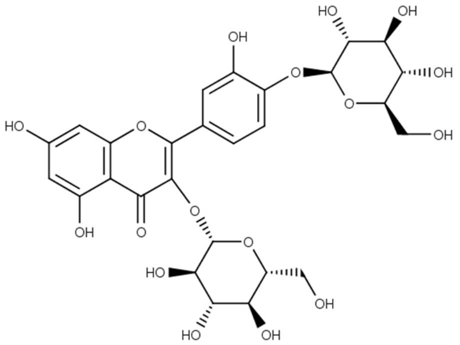
quercetin 3,7-di-*O*-β-D-glucopyranoside	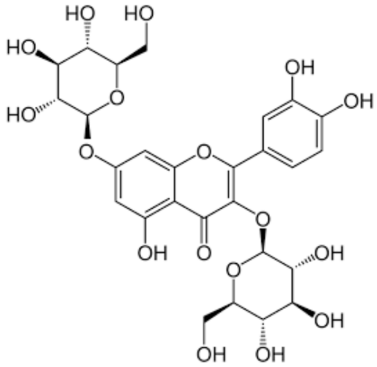
rutin	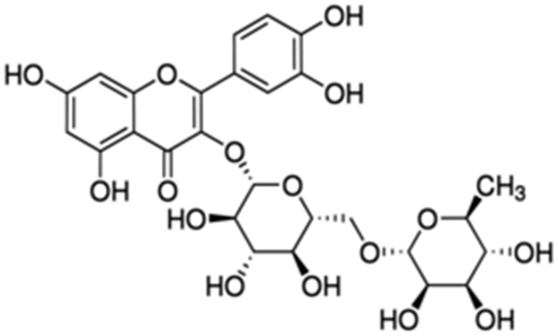
myricetin	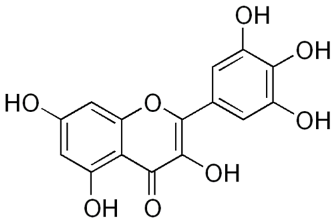
naringenin	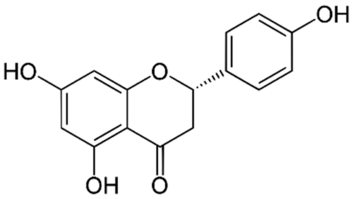

taxifolin	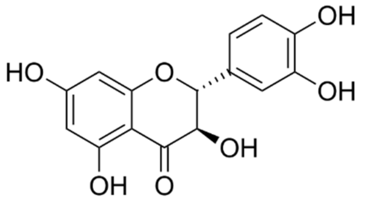
tamarixetin	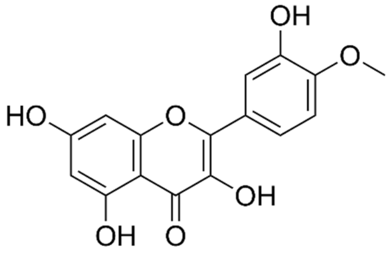
isorhamnetin	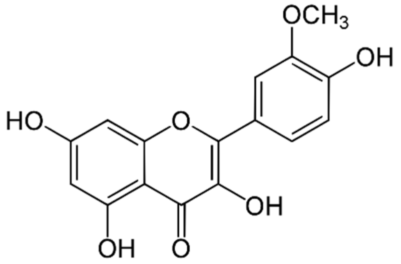
isorhamnetin 3-*O*-β-D-glucopyranoside	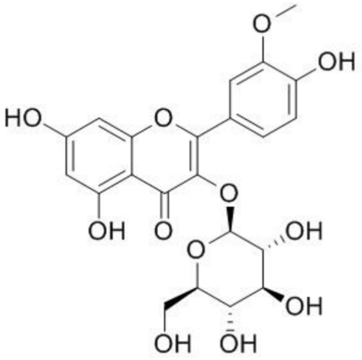
isorhamnetin 3,4′-di-*O*-glucoside	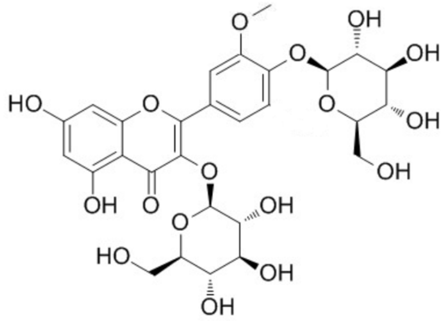

isorhamnetin 3,7-di-*O*-β-D-glucopyranoside	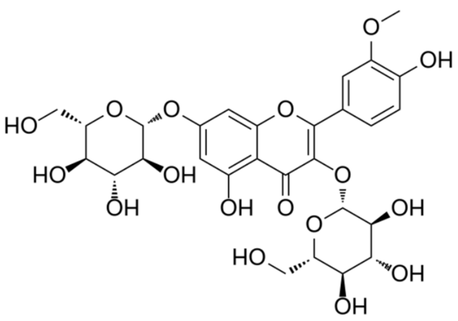
delphinidin 3-*O*-glucoside	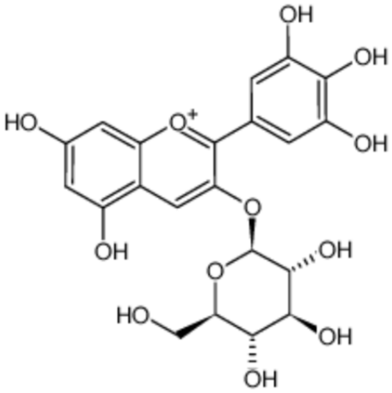
delphinidin 3,5-di-*O*-glucoside	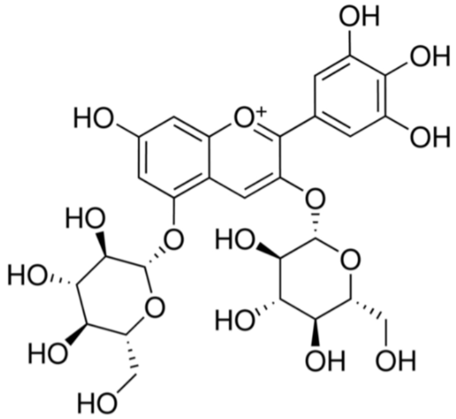
delphinidin 3,7-di-*O*-glucoside	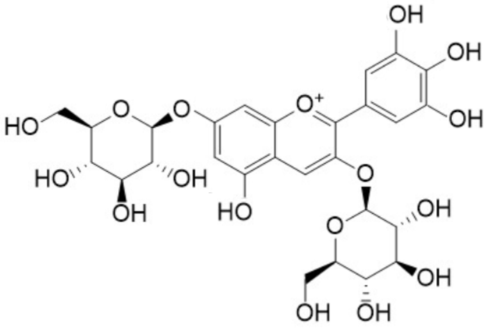

delphinidin 3-*O*-β-rutinosides	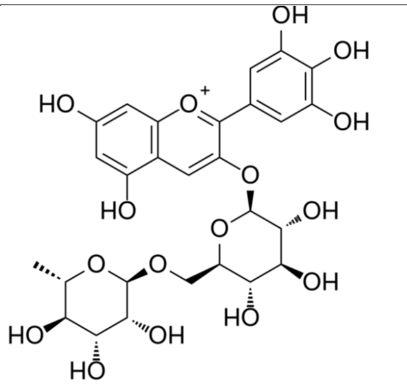
delphinidin 3-*O*-β-glucoside-5-*O*-(6-*O*-malonyl-β-glucoside)	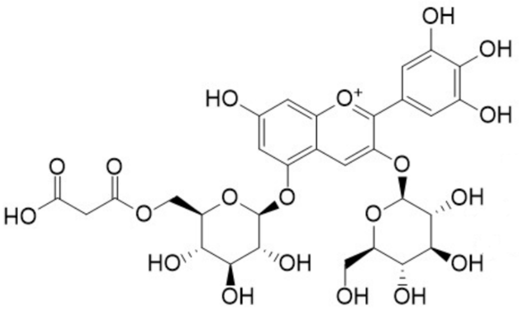
petunidin 3-*O*-(6-*O*-malonyl-β-glucoside)-7-*O*-(6-*O*-malonyl-β-glucoside)	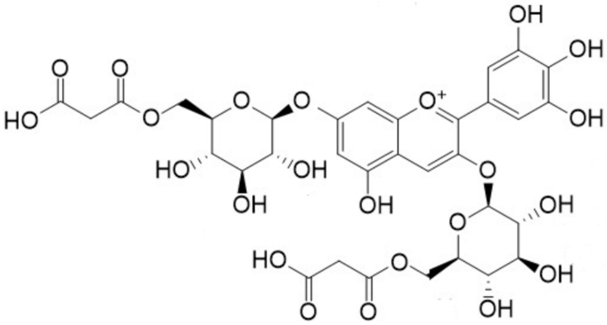
malvidin 3-*O*-glucoside	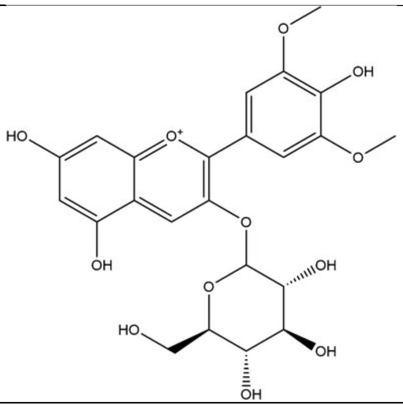

malvidin 3,5-di-*O*-glucoside	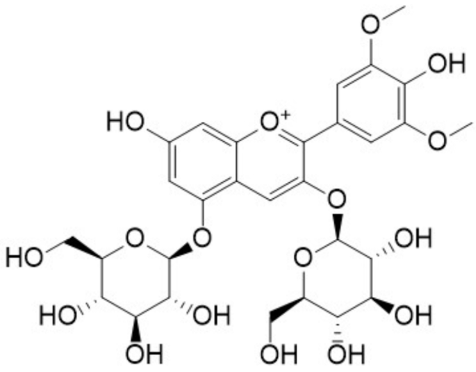
malvidin 3-*O*-(6-*O*-malonyl-β-glucoside)-7-*O*-(6-*O*-malonyl-β-glucoside)	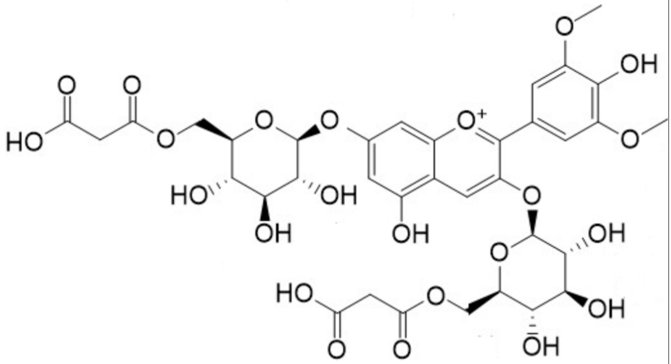
petunidin 3-*O*-glucoside	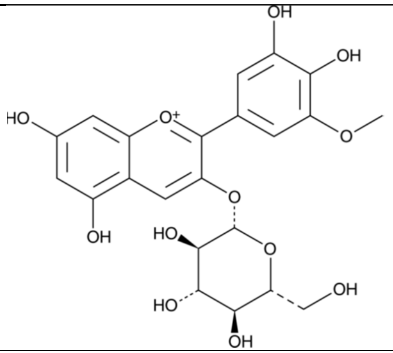
petunidin 3-*O*-β-rutinosides	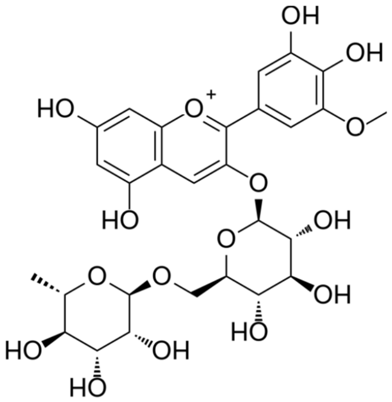

petunidin 3,7-di-*O*-β-glucosides	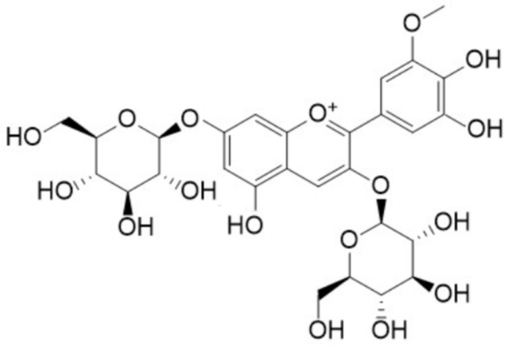
petunidin 3-*O*-(6″-p-coumaroyl-glucoside)	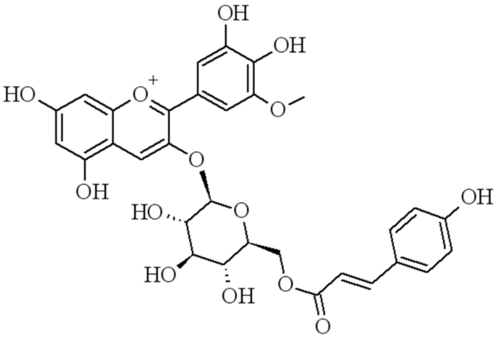
peonidin 3,5-*O*-diglucoside	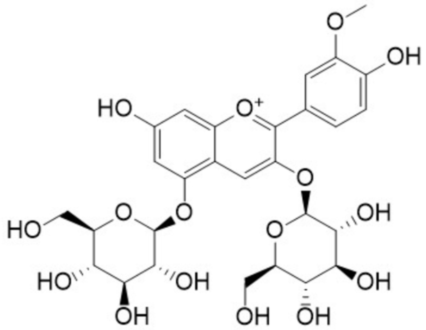
peonidin 3,7-di-*O*-β-glucosides	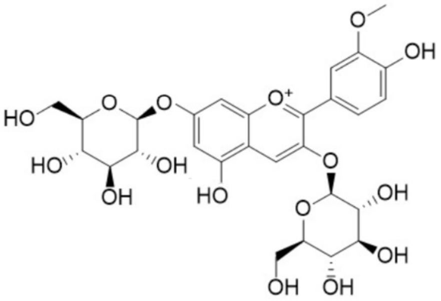
cyanidin	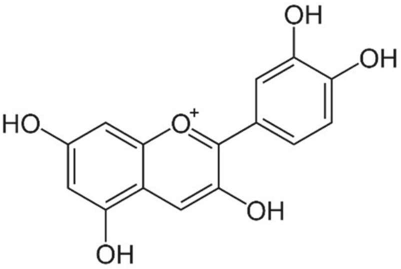

3-(*R*)-3-β-D-glucopyranosyloxybutanolide	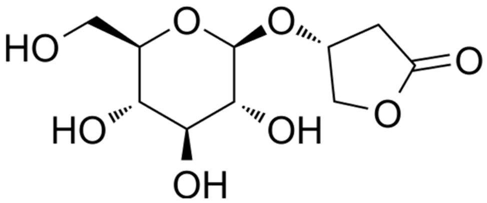
3-(*S*)-3-β-D-glucopyranosyloxybutanolide	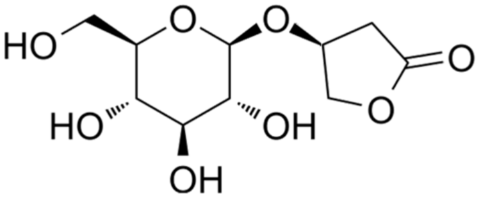
ferulic acid	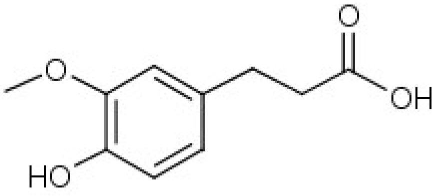
cinnamic acid	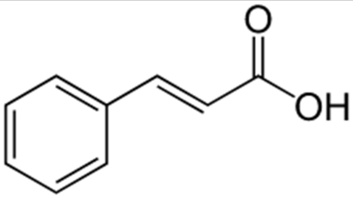
ellagic acid	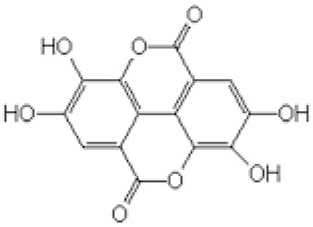
catechin	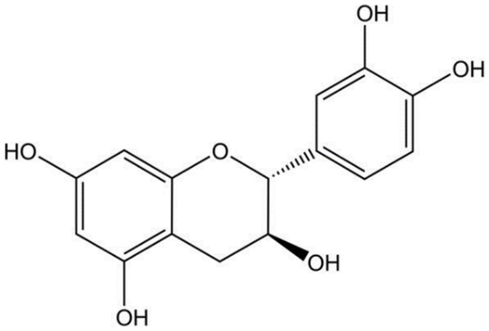

epicatechin	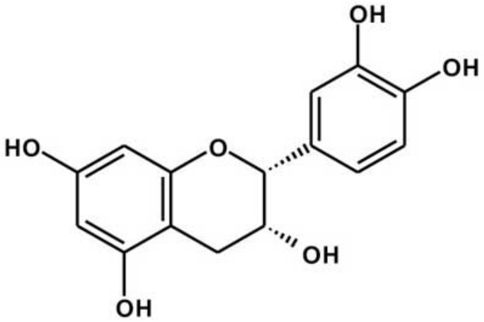
vitamin C	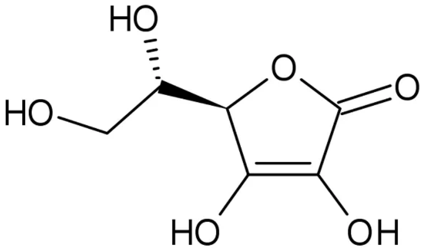
caftaric acid	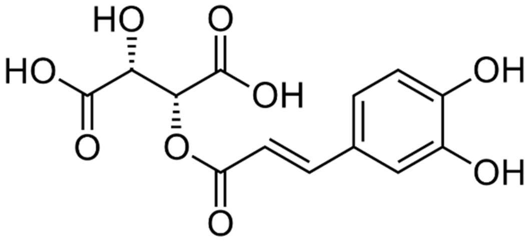
apigenin	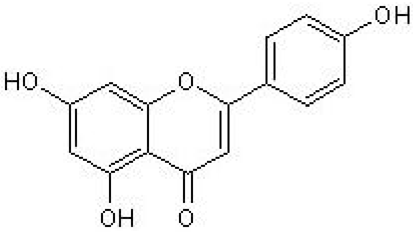
luteolin	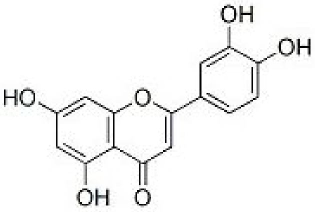
fisetin	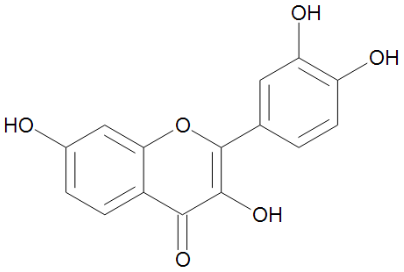

hesperitin	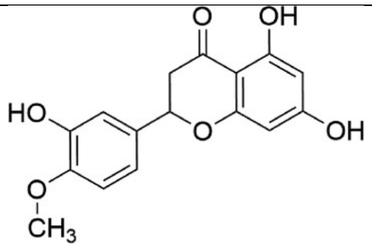
